# Effects of density and fire on the vital rates and population growth of a perennial goldenaster

**DOI:** 10.1093/aobpla/plt041

**Published:** 2013-09-09

**Authors:** Elise S. Gornish

**Affiliations:** 1 Department of Biological Science, Florida State University, Tallahassee, FL 32306-4295, USA; 2 Present address: Department of Plant Sciences, University of California, Davis, Davis, CA 9561, USA

**Keywords:** Demography, disturbance, matrix model, *Pityopsis aspera*, regression-design LTRE.

## Abstract

Intraspecific density effects are generally associated with other factors, like disturbance. Therefore, the ways in which density effects might interact with disturbance to modify the relationships between vital rates and population growth must be understood. I quantified the effects of density on the life-history stages of the perennial composite *Pityopsis aspera* over 3 years, the span of which included years in which fire did and did not occur. In an experimental study, I estimated the survival, growth and reproduction for shoots in plots established across a natural range of densities in Florida, USA. In a novel analysis, a regression-design life-table response experiment was used to determine which transitions were associated with density, how they contributed to differences in estimated population growth rates and how this relationship differed as a result of fire. The shape of the relationship between population growth rate (*λ*) and density was modified by fire, primarily as a result of contributions from adult flowering stasis and survival, and first-year survival probabilities. Fire modified and even reversed the effect of extreme densities on adult flowering stasis and survival and of first-year survival, resulting in more positive contributions from these transitions to *λ* at the lowest and highest density values. These results demonstrate the first application of a regression-design life-table response experiment to elucidating the interactive effects of density and fire. They highlight the utility of this approach for both capturing the complex dynamics of populations and establishing a means of determining how vital rates might contribute to differences in demography across densities.

## Abstract (precis)

In a novel analysis, a regression-design life-table response experiment was used to determine how the interaction of fire and density affected vital rates of the perennial composite *Pityopsis aspera*, and ultimately how these changes in vital rates contributed to differences in estimated population growth rates.

## Introduction

The regulatory causes and effect of within-population density are among the most important and most documented topics in ecology. Differences in population density across sites are expected to be caused by a variety of factors, including differences in habitat quality (Holt 1987), settlement costs (Greene and Stamps 2001) and dispersal (Amarasekare 2004). As an effect, density often plays a role in the modification of resources and both intra- and interspecific interactions (see e.g. Hixon *et al.* 2002; Sibly *et al.* 2005). As intraspecific density increases, competition for limiting resources becomes more intense (Antonovics and Levin 1980; Miller 1996), leading to decreases in the size and often the number of individuals (e.g. the self-thinning rule; Hutchings 1983). At low densities, phenomena like Allee effects (Feldman and Morris 2011) can reduce vital rates indirectly by, for example, reducing seed production (Davis *et al.* 2004).

Clearly, however, density effects do not operate independently of other drivers of population dynamics (Dahlgren and Ehrlén 2009). For example, disturbance, which involves the irregular alteration of resource availability, can result in direct modifications of vital rates and biomass (Grime 1979), as well as indirect effects through changes in density (see e.g. Casper 1996). A disturbance that removes vegetation can open up space that the surviving plants can take advantage of through higher rates of tiller production (Fetcher and Shaver 1983). Alternatively, disturbance can exacerbate density effects. For example, a disturbance that reduces an already small plant stand can cause the stand to attract fewer pollinators per plant (see e.g. Ghazoul *et al.* 2012). Investigating the way in which density and disturbance interact can highlight how multiple ecological factors operate at the individual and population levels.

Fire, an episodic terrestrial disturbance, can affect organisms both physically and chemically (Bond and van Wilgen 1996). Obviously, fire often has a negative direct effect by burning above-ground biomass, but it can also increase nutrient mobility and availability by burning litter, depositing ash and increasing microbial activity and nitrogen fixation (Christensen 1981; Hulbert 1988) and often has positive effects on germination and flowering (Daubenmire 1968) and population growth (see e.g. Lesica 1999). Although the landscape-scale effects of fire are well known, studies investigating demographic responses to fire are still relatively uncommon (but see Menges and Dolan 1998; Liu *et al.* 2005; Lloyd *et al.* 2005; Evans *et al.* 2010). Fire has been shown to have dissimilar effects on different life-history stages, even to the point of having negative effects on some stages and positive effects on others (see e.g. Schemske *et al.* 1994; Kesler *et al.* 2008).

I investigated how fire modified the relationship between density and population growth rate in a common understorey herb. Because density dependence can operate differently on different life stages (Hackney and McGraw 2001; Rey *et al.* 2004) and results in non-linear relationships between life-stage transition probabilities and population growth (de Kroon *et al.* 2000), these relationships can only be determined by assessment of vital-rate responses to a range of densities (Stokes *et al.* 2004). Life-table response experiments (LTREs) have been successfully, but only infrequently, used to look at the relationship between density, life-history traits and population growth rate (Oli *et al.* 2001) and would be an appropriate approach to quantify density effects. But, simple LTREs can be ineffective in capturing the relationship between population growth rate (*λ*) and density when density varies continuously rather than being categorized simply as ‘high’ and ‘low’. I therefore used a regression-design LTRE (Caswell 1996), which can be a more informative way of investigating how a realistic range of treatment values affects *λ* through differential effects on life-history stages (e.g. Kesler *et al.* 2008). This approach allows the estimation of density effects on the asymptotic growth rate of a density-independent population, within a framework that is familiar to most modellers. Although plant density is often a response to an underlying environmental factor, using a regression-design LTRE to assess how vital rates change across a natural gradient of plant frequency is useful for understanding biotic processes that ultimately arise from interacting abiotic factors.

I predicted that population growth rates should decrease with increasing density in both a year in which fire was absent (hereafter referred to as a non-fire year) and a year immediately following a fire (hereafter referred to as a fire year), as a result of competition for limiting resources or increased physical interference among individuals (Ban *et al*. 2009). Although life stages can differ in the magnitude of density effects (Grant 1998; Buckley and Metcalf 2005), increasing density is expected to have a generally negative effect on vital rates because of concomitant reductions in resource availability (Jesson *et al.* 2000; Levine *et al.* 2004). Second, I predicted that non-fire and fire years should differ in the way in which vital rates contributed to *λ* across densities. The effects of fire, which could be density dependent (see e.g. Linke-Gamenick *et al.* 1999), potentially because of differences in the life-history strategies employed to manage the effects of these two factors (Gross *et al.* 1998), have been shown to have significant positive as well as negative effects on vital rates (by e.g. Kaye *et al.* 2001). The interactive effects of density and fire are therefore expected to be non-additive.

## Methods

### Data collection

**Figure 1.**
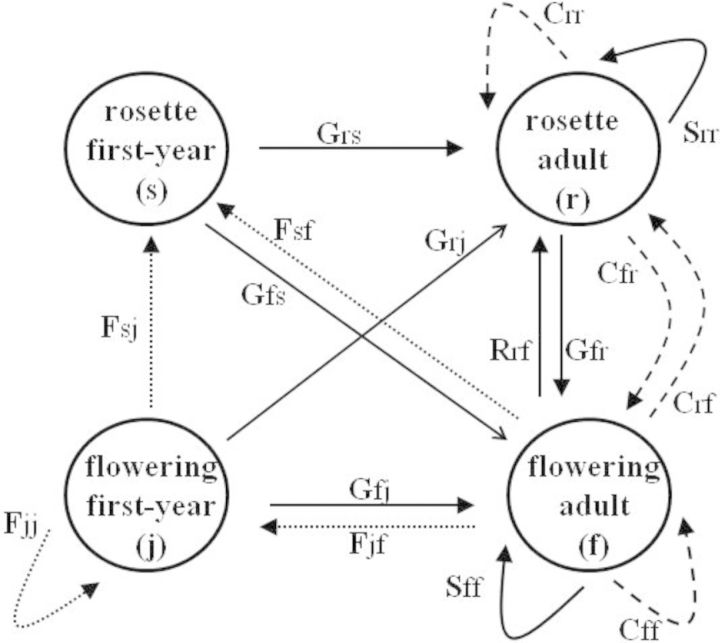
Irreducible life cycle of *P. aspera*, with four stages (s, rosette first-year; j, flowering first-year; r, rosette adult; and f, flowering adult). Transitions between stages represent G, growth; S, stasis; and R, retrogression, all represented by solid arrows. F, sexual reproduction, is represented by a dotted arrow; C, clonal reproduction, is represented by a dashed arrow.

The native perennial composite *Pityopsis aspera* var. *aspera* (Asteraceae), a goldenaster commonly known as pineland silkgrass, is a herbaceous dicot common in xeric sandhill habitats (Myers and Ewel 1990) in northern Florida and south Georgia. It is self-incompatible (Bowers 1972), reproducing both vegetatively and sexually. Studies have shown that fire increases flowering (Gowe and Brewer 2005) and reduces the average shoot size (Brewer and Platt 1994*a*) of individuals in the genus. The life cycle of *P. aspera* can be divided into four distinct stages based on age, survival and flowering probabilities (Fig. 1): rosette first-year (shoots in their first year of life that have small, upright leaves in a rosette form), flowering first-year (shoots in their first year of life that also produce flower heads), rosette adult (larger, prostrate rosettes that do not produce flowers) and flowering adult (adults that also produce flower heads). A 2-year field germination bag test suggests that this species does not maintain a seed bank (E. Gornish, unpubl. data), so no seed stage is included in the life cycle (e.g. Caswell 2001). First-year shoots have significantly lower survival than do adult plants. First-year plants must become adults after 1 year (undergo growth; although they could remain the same size). Every year, rosette adults must either remain in their stage class (undergo stasis) or become flowering adult individuals (undergo growth). Every year, flowering adult individuals can either remain in their stage class (undergo stasis) or become rosette adults (undergo retrogression). Only adult plants can reproduce asexually. When the study was initiated, differentiation between shoots produced sexually vs. asexually was impossible. As a result, sexually and asexually produced shoots were treated as the same in my models. Unpublished data (currently in review) suggest that shoots produced from asexual reproduction have similar survival and fecundity probabilities as sexually produced shoots.

Nine 1-m^2^ plots were established at the Tall Timbers Research Station, FL, USA, in August 2009 in areas with differing densities of *P. aspera*. The habitat is a mixed loblolly–shortleaf pine forest (Reid *et al.* 2012) with a humid, subtropical climate. Precipitation at the site averages 100 cm per year, and the average daytime temperature is 20 °C. Fire, which was historically caused by lightning every 2 to 8 years (Christensen 1981), is now regulated by surface-fire prescribed burning. Forests maintained by Tall Timbers Research Station have been frequently burned (every 1–2 years, on average) since ca. 1900.

The study plots were established in random locations (using a random coordinate generator) within a typical population of *P. aspera*, which covered ∼40 m^2^. The plots included a natural range of nine densities (which captured minimum and maximum density; 71, 77, 91, 132, 138, 166, 179, 196 and 206 shoots m^−2^) for a total of 1259 shoots mapped and followed. All shoots in each plot were marked with a metal bird tag with a unique ID number. Each August, the fate of each marked shoot was recorded. Seedlings were identified during the fall and marked with a toothpick. Plots were separated by a minimum of 8 m. A multiple regression suggested that percentage soil moisture and soil organic material (measuring using combustion methods of soil cores in the laboratory) increased with density (non-significantly) and that soil pH [measured *in situ* using a Fieldscout SoilStick pH meter (Spectrum Technologies, Inc.)] decreased with density (*P* = 0.03). The absence of stronger relationships could be due to the lack of power or the presence of outliers **[see****Supporting Information****]**. A Durbin–Watson test did not detect the presence of spatial autocorrelation on density (one-tailed test: DW = 2.286, *P* = 0.6403); however, possible differences in non-target community aggregations could have effects on *P. aspera* population dynamics (Rayburn and Schupp 2013). Relative densities of plots did not change during the 3 years of the study. Life stage (rosette first-year, flowering first-year, rosette adult and flowering adult) and flower-head number were recorded for each shoot to provide estimates of survival, growth and reproduction.

Adequate sampling across densities required a trade-off with sampling intensity (the number of shoots sampled within a year; Doak *et al.* 2005), and thus could increase uncertainty around estimates of vital-rate means and possibly inflate population growth rate (Caswell 2001; Doak *et al.* 2005). However, the deterministic models used in this study are more robust to limitations of sampling intensity than are stochastic models (Doak *et al.* 2005). Moreover, preliminary studies of *P. aspera* suggest high adult survival rates, which have been shown to reduce bias in estimating population growth rate (e.g. Fiske *et al.* 2008).

### Population demography

All shoots were censused three times (2009, 2010 and 2011) in late August after flower heads had developed. Each year, 30 flower heads were collected from flowering shoots near, but outside of, each plot for flower-number estimates. The annual population fecundities of flowering shoots were estimated as *F_i_* = (average number of flowers per flower head at the density in which type *i* plant is found) × (number of flower heads per shoot) × (average germination at the density in which type *i* plant is found). The contribution of flowering shoots (both first-year and adult) to first-year stage classes was calculated as the proportion of flowers each flowering shoot contributed to a plot in year *t* × the number of newly germinated plants documented for year *t* + 1.

Because following seeds in the field was not feasible, I estimated germination rate by collecting 100 seeds from flowering shoots near (within 2 m; thereby sampling from shoots experiencing similar density effects) but not in each plot in October of each year and germinating them in a growth chamber; ungerminated seeds were subjected to a tetrazolium test for viability. This allowed me to both estimate average germination rates for each density and estimate ratios of first-year stage (rosette vs. flowering) germination for each density. The germination rates of seeds from flowering first-year and flowering adult shoots were similar.

Annual stage-based population-projection matrices were parameterized with the transition probabilities of each shoot in each plot, which described the transformation of the number of shoots from one year to the next over 3 years (producing two matrices per density) **[see** **Supporting Information****]**. Possible transitions were growth, stasis and retrogression. Matrix values for asexual reproduction and sexual reproduction were also estimated. Matrix values for asexual reproduction were estimated by counting new shoots in the plots and assuming that they were ramets from the nearest marked plant. Emerging first-years were differentiated from emerging ramets by the presence of cotyledons.

### Analysis

I conducted two separate analyses. First, I conducted analysis of covariance (ANCOVA) to determine whether the categorical factor fire (subjected to fire and not subjected to fire in a year), the continuous factor density or the interaction between the two contributed to differences in *λ* and vital rates. Response variables were transformed for normality when necessary, and all analyses were conducted in R version 2.15.

Second, I used a regression-design LTRE. Two projection matrices were created for each of the nine plots: one describing transition probabilities between 2009 and 2010 and one describing transition probabilities between 2010 and 2011. No prescribed burning was applied to the experimental plots between August 2009 and August 2010, so these matrices will be referred to as matrices in the non-fire year. In mid-April 2011, a low-intensity strip-head burn was applied to the experimental plots, so matrices describing the dynamics between 2010 and 2011 will be referred to as matrices in the fire year.

An LTRE is a retrospective decomposition analysis that quantifies the effects of experimental treatments on population growth rate (Caswell 2001). Because the effects of matrix elements on *λ* are often measured in different units, an LTRE translates these effects into contributions to *λ*, allowing direct comparison among matrix elements and vital rates. For the study reported here, where the treatment (density) varied along a continuum, a regression-design LTRE (with plot as the experimental unit) was particularly useful in investigating the relationship between density and *λ*.

For each matrix, *λ* was determined as the asymptotic rate at which a population grew at the stable stage distribution and calculated as the largest eigenvalue of the matrix. A Taylor's series expansion (Alvarez-Buylla and Slatkin 1993) was used to estimate approximate 95 % confidence intervals for *λ*. The sensitivity demonstrates how *λ* changes as matrix elements change and was calculated as (Caswell 2001)
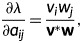

where * denotes the complex conjugate transpose, **w** is the stable age distribution calculated as the right eigenvector of the matrix and **v** is the reproductive value calculated as the left eigenvector.

Matrix elements as a function of density were expressed by means of a non-parametric regression model. I used a locally weighted polynomial regression running line smoother (Hastie and Tibshirani 1990) determined with the *lowess* function in the stats package in R (http://www.R-project.org/) for descriptive purposes. Together, the sensitivity and the slope of the regression are used to conduct a regression-design LTRE, which decomposes the effect of density (*x*) on *λ* (d*λ*/d*x*) into contributions from each matrix element:
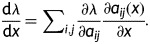



In addition to looking at the contributions of individual matrix elements, I also assessed how several lower-level vital rates contributed to density effects on *λ* (d*λ*/d*x*). This included survival of each stage class, sexual reproduction of flowering first-year and flowering adult stage classes, and asexual reproduction from adult stage classes.

## Results

### ANCOVA: vital-rate response to density and fire

**Figure 2.**
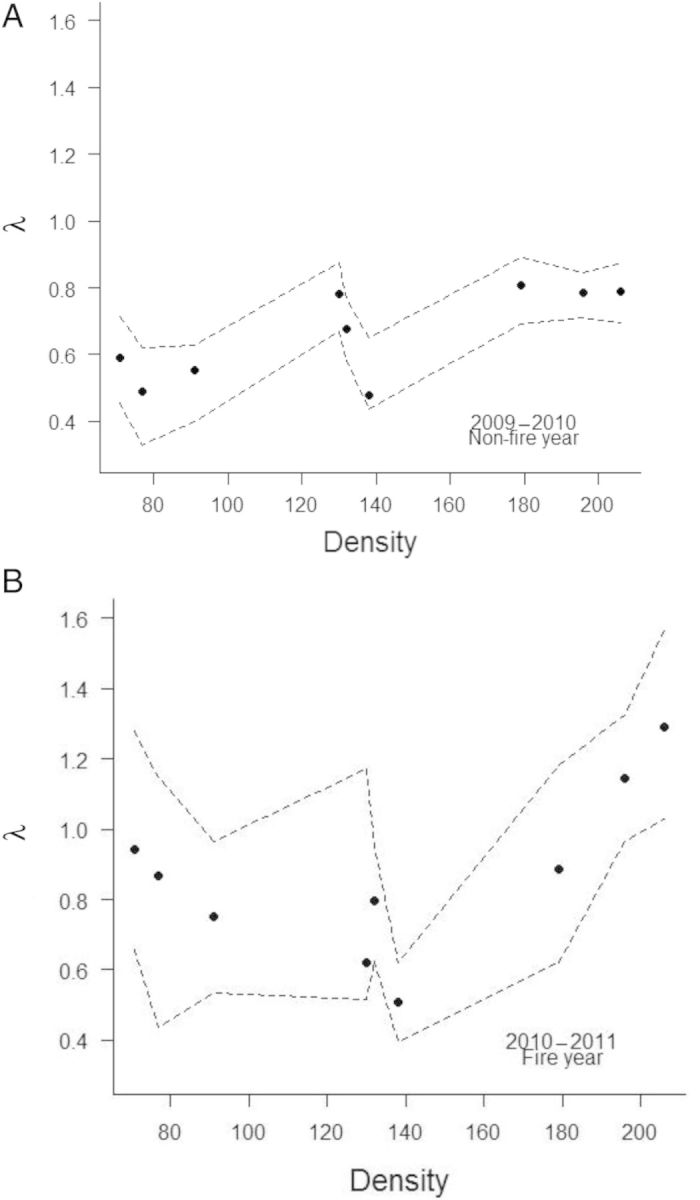
Population growth rate (*λ*) as a function of density in (A) non-fire and (B) fire years. Dotted lines indicate upper and lower 95 % confidence intervals.

Population growth rate had different relationships with density in the non-fire and fire years (Fig. 2); however, the interactive effect was not significant, likely due to non-linear relationships between density and *λ*. In the non-fire year, *λ* had a positive, linear relationship with density (mean low density *λ* = 0.53, mean medium density *λ* = 0.65, mean high density *λ* = 0.79; *y* = 0.002*x* + 0.38; *R*^2^ = 0.57, *P* = 0.02). In the fire year, the relationship was curvilinear (*y* = 0.34*x*^2^ + 0.55*x* + 0.87; *R*^2^ = 0.901, *P* = 0.008), where *λ* was highest at the most extreme density values (196 density *λ* = 1.14, 206 density *λ* = 1.29; Fig. 2B). The values of *λ* were higher in the fire year (mean non-fire year *λ* = 0.66, mean fire year *λ* = 0.87; *F*_1,14_ = 6.43, *P* = 0.02). Density also contributed to differences in *λ* (mean low density *λ* = 0.70, mean high density *λ* = 0.95; *F*_1,14_ = 6.67, *P* = 0.02).

Vital rates had dissimilar relationships with density in both the non-fire and fire years. Fecundity was highest at intermediate density in the non-fire year (Table 1). Fecundities of plants that withstood the burning had a positive relationship with density in the fire year and were, in general, lower in the fire than in the non-fire year (fire × density effect *F*_1,14_ = 6.3, *P* = 0.02). In the non-fire year, flowering first-year survival and flowering adult survival were positively related to density and were higher, on average, than those in the fire year. Neither first-year survival nor flowering adult survival appeared to be related to density in the fire year (fire × density effect *F*_1,14_ = 4.46, *P* = 0.05 and *F*_1,14_ = 76.90, *P* < 0.001, respectively). Percentage rosette adult survival was positively related to density in both the non-fire and fire years (*F*_1,14_ = 4.94, *P* = 0.04) and was higher, on average, in the fire year (fire × density effect *F*_1,14_ = 1.33, *P* = 0.27). Rosette adult asexual reproduction was negatively related to density in the non-fire year but positively related in the fire year (fire × density effect *F*_1,14_ = 9.11, *P* = 0.009). Although flowering adult asexual reproduction was slightly higher, on average, in the non-fire than in the fire year, this vital rate was not related to density in either year (fire × density effect *F*_1,14_ = 2.31, *P* = 0.15; Table 1).

**Table 1.** Average vital rates across densities in years in which the experimental shoots were not subjected to fire (2009–10) and in which they were (2010–11). Fecundity is number of seeds produced per shoot; survival and asexual reproduction values are average percentages.

	Density (number of shoots per unit area)								
	71	77	91	130	132	138	179	196	206
First-year fecundity									
Non-fire	96	33	336	361	114	113	197	50	180
Fire	52	0	24	25	32	0	11	76	24
Adult fecundity									
Non-fire	427	503	670	692	743	424	552	386	490
Fire	280	19	265	189	344	244	160	140	393
Rosette first-year survival									
Non-fire	17	19.4	25	34.4	17.3	34	48.5	50	66.7
Fire	23	5.2	5.7	5.5	13.7	3.6	4.8	61.4	33.3
Flowering first-year survival									
Non-fire	25	42.9	50	61.9	34.3	47.4	45.8	43.5	72.7
Fire	19.2	0	11.1	15	30.4	5.6	12.1	70.6	100
Rosette adult survival									
Non-fire	52.5	42.1	54.5	53.9	66.7	40.7	59.6	71.3	65
Fire	69.6	44.6	32	46.5	46.5	46.2	58.7	57.8	64.8
Flowering adult survival									
Non-fire	56.1	38.1	52.6	70	62.7	57.6	62.3	63.3	63.7
Fire	33.3	75	25	27.8	25	36.5	38.1	38.9	66.6
Rosette asexual reproduction									
Non-fire	26.1	31.1	31.8	25.8	19.8	25.4	17.5	16	20
Fire	7.7	0	1.6	2.1	7.5	2.1	4.3	4	5.6
Flowering asexual reproduction									
Non-fire	37.0	26.2	22.4	17.1	15.5	23.5	20	24.5	20.2
Fire	0	25	0	2.7	13	0	0	11.1	5.5

### Regression-design LTRE

**Figure 3.**
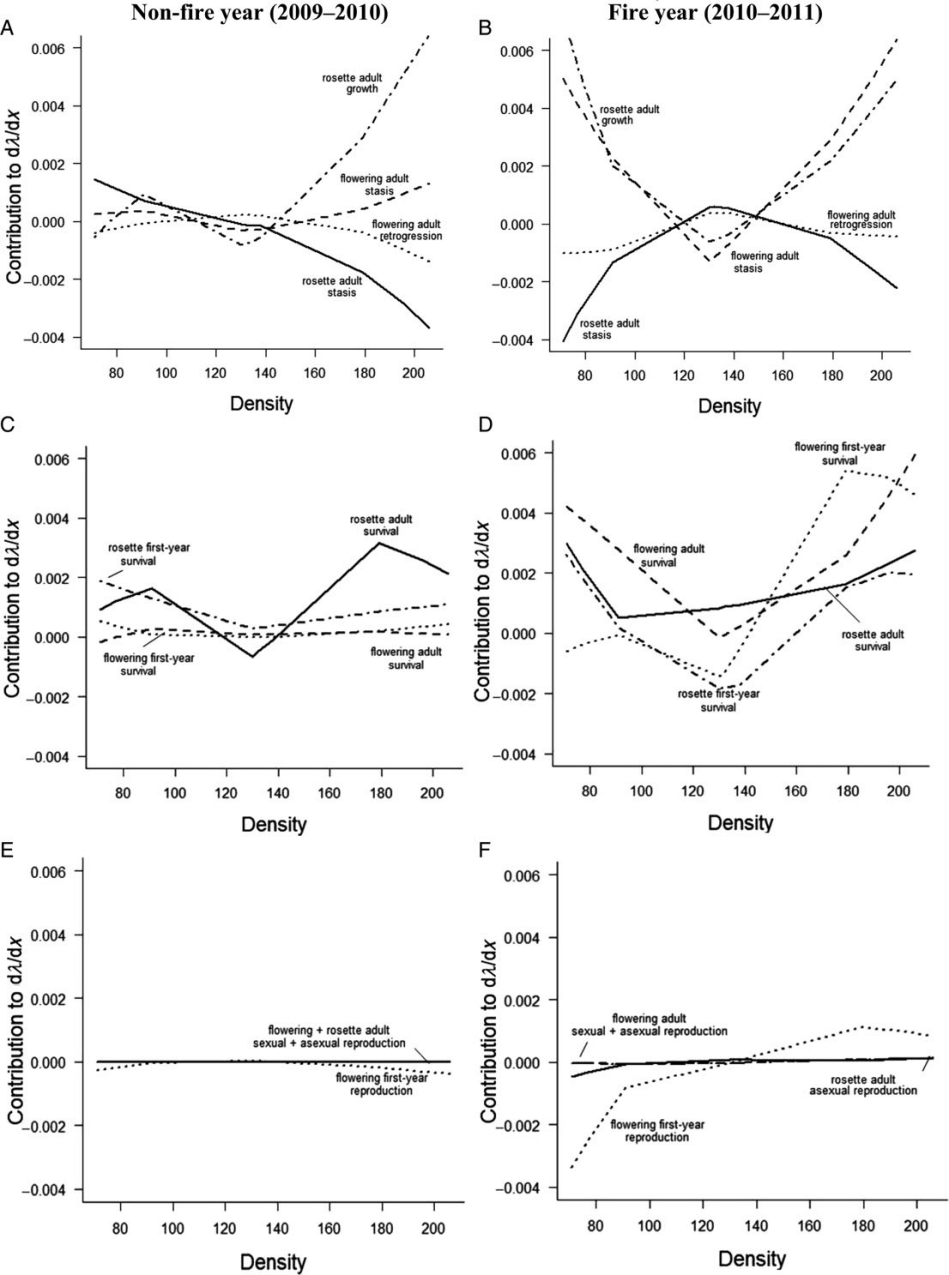
Life-table response experiment contribution of matrix elements (A, B) and vital rates (C–F) to the effect of density on population growth rate in the non-fire year (2009–10) and in the fire year (2010–11).

In the non-fire year, the contributions of flowering adult retrogression and stasis to density effects on *λ* (d*λ*/d*x*) demonstrated opposing unimodal relationships with density (Fig. 3A). This pattern was maintained in the fire year for flowering adult retrogression, whereas rosette adult stasis contributed more negatively to d*λ*/d*x* at extreme values in density in the presence of fire (Fig. 3B). In the absence of fire, the contribution of rosette adult stasis to d*λ*/d*x* became more negative with increasing density, contrary to the contribution of rosette adult growth, which became much more positive with increasing density. In the presence of fire, the contributions of both of these vital rates to d*λ*/d*x* demonstrated a more distinct unimodal relationship (Fig. 3B).

In the non-fire year, first-year and flowering adult survival made similar, non-significant contributions to d*λ*/d*x* across density (Fig. 3C). In the fire year, contributions from the survival of all life stages to d*λ*/d*x* demonstrated unimodal relationships with density. Survival of the two adult life stages contributed positively to d*λ*/d*x* across densities, whereas survival of the two first-year life stages generally contributed positively to d*λ*/d*x* only at the lowest and highest values of density (Fig. 3D).

In the non-fire year, sexual and asexual reproduction of all life stages did not appear to contribute significantly to d*λ*/d*x* across density (Fig. 3E). In contrast, in the fire year, the contribution of flowering first-year sexual reproduction had a positive relationship with density (Fig. 3F).

## Discussion

Density effects are pervasive in ecology and can be especially important for species with complex life cycles, in which density effects at one stage can have implications for other stages (Johnson 2008). Although a well-developed literature addresses incorporation of density dependence into matrix models (Logofet 1993; Dennis *et al.* 1995; Caswell 2001), the methods often require more complex calculations and, more importantly, additional data that might not be regularly collected by demographers. I used a regression-design LTRE, which addressed both of these issues, to examine the simultaneous effects of density and fire on demography. This approach allowed the detection of complex interactions between density and fire within and across life stages (Lesica 1999; Goldberg *et al.* 2001), supporting expectations of non-linear plant response to changes in neighbour density and disturbance (Table 1; Goldberg 1987).

In general, *λ* is expected to have a negative relationship with density because competition among clonal ramets of *P. aspera* is expected to reduce plant performance as density increases (Thomas and Dale 1975). My results do not support this expectation, suggesting that the underlying factors creating a high-quality environment also lead to high densities (and, often, high diversity, e.g. Carpenter 2005), and override density effects on the population; sites where plants grow well will result in higher densities of plants. Alternatively, an unmeasured extreme weather event such as a drought can equal or supersede the effect of density (e.g. Brewer 2006). To be assured that density can be definitively identified as an actual cause of differences in demographic rates, it must be experimentally manipulated (Fowler *et al.* 2006) for several years or environmental conditions should be shown to be the same across observed density gradients. Alternatively, the size structure of the population may have changed across densities (Chapin 1991), modifying competitive interactions through differences in resource uptake among individuals (Goldberg 1990), thereby reducing density effects on *λ*.

As predicted, the shape of the relationship between density and *λ* in a fire year was found to differ from that in a non-fire year (Fig. 2), probably because of differences in the factors driving within-population density effects (Antonovics 1976). In the non-fire year, the relationship between density and *λ* was positive, and in the fire year, the relationship was unimodal. Environmental factors, like fire, have been shown to modify the importance of competition, for example, in driving population dynamics in high-density centre areas and through Allee effects at low-density edge areas (Holt *et al*. 2005). The presence of fire has also been shown to indirectly affect density through effects on sexual and clonal reproduction (e.g. Brewer 2006, 2008). The non-intuitive relationship among *λ*, density and fire suggests that experimental tests of disturbance effects on *λ* at a single density could be inadequate and misleading. Considerations of time since last fire (e.g. Caswell 2010) and seasonality of fire (e.g. Brewer and Platt 1994*b*) would also be valuable for assessing complex life-history effects.

The observational nature of this study makes it difficult to tease apart how environmental factors could have modified the effects of fire on *P. aspera*. Also, because fire covaried with year, an unmeasured year effect could also be responsible for changes in vital rates between the two study periods. Using 3 years of data to parameterize my population-projection matrices restricts my ability to adequately assess across-year variation and could result in an overestimation of *λ* (some models suggest that >20 years of data are necessary to minimize parameter uncertainty: Evans *et al.* 2010). Indeed, plant performance can be influenced by time and by time × density interactions (e.g. Rayburn and Schupp 2013). For example, climatic anomalies present in only 1 year of the study could have overshadowed fire effects on *P. aspera* vital rates directly (Evans *et al.* 2010) or indirectly through modifications to dominant community members (e.g. Arnone *et al.* 2011).

However, my results agree with other studies of plant demographic responses to fire in sandhill habitats (Evans *et al.* 2010; Weekley and Mendes 2012), which demonstrate the dominant effect of fire compared with other environmental factors in fire-controlled systems (Menges *et al.* 2011). Year effects on *P. aspera* were also expected to be small during the tenure of this study. Both precipitation and temperature (data obtained from the Southeast Regional Climate Center: http://www.sercc.com) demonstrated similar means and variances between 2008 (to account for carryover effects; Sherry *et al.* 2012) and 2011. Moreover, long-term simulations suggest that the effects of fire can regularly supersede year effects, especially in fire-dominated systems (Wang *et al.* 2007). All this suggests that my results likely highlight very real demographic effects of fire; however, experimental manipulations (Fowler *et al.* 2006) would be necessary to completely separate the effects of fire from the effects of environmental conditions across years.

The regression-design LTRE approach was useful for identifying the particular vital rates that contributed the most to differences in d*λ*/d*x*. For example, in both the fire year and the non-fire year, differences in sexual and asexual reproduction across densities (Table 1) suggest that both fire and density play large roles in modifying the effects of reproduction and density on *λ*. Results of the regression-design LTRE, however, revealed the complex and mostly negligible contributions of reproduction to differences in d*λ*/d*x* (Fig. 3), suggesting that *λ* can have a much lower sensitivity to fecundity than other vital rates at density extremes (Feldman and Morris 2011). Similarly, an effect of density on reproductive LTRE contributions for flowering first-year fecundity was found only in the fire year, possibly as a response to higher availability of resources resulting from an overall reduction in adult asexual reproduction at higher densities (Gadgil and Solbrig 1972). Alternatively, higher allocation to first-year sexual reproduction may be acting to facilitate ‘escape’ from the intense competitive pressure at high densities (Ogden 1974; Loehle 1987), resulting in larger contributions to d*λ*/d*x*.

The regression-design LTRE was also useful for identifying life-history traits that were affected by density and fire but did not significantly affect *λ*. Identifying interactions like these is important for understanding underlying mechanisms driving the relationship between frequency, fire and population growth. For example, the increase in overall survival of first-years with density (Table 1) was not manifest in the LTRE analysis in the non-fire year. Perhaps a large number of germinated seeds were surviving to the first-year stage (Hartnett and Bazzaz 1985) in the non-fire year, thereby reducing the importance of the per capita survival of the first-years to population growth. First-year survival was also higher in the non-fire year, potentially as a result of greater secondary growth (Hobbs and Mooney 1985), but did not appear to contribute to changes in *λ* across density between 2009 and 2010. These results highlight the importance of looking at individual matrix elements, vital rates and population-level treatment effects in developing a comprehensive understanding of the relationship between treatments and demography.

## Conclusions

Biotic factors like density have been predicted to have larger effects on older stage classes of plants by constraining abundance (Levine *et al.* 2004) than do abiotic factors, which are predicted to have larger effects on early stage classes by driving seedling survival and growth rates (Truscott *et al.* 2008) and affecting recruitment (Dalgleish *et al.* 2010). Unexpectedly, I found that density affected vital-rate contributions across stage classes, except for those related to reproduction. Further, the effects of density changed with fire; generally, fire appeared to increase the overall contribution of transition probabilities to d*λ*/d*x* (except for rosette adult stasis), but only at extreme values of density. Overall, interspecific competition was probably attenuated by fire through the reduction of potentially less well-adapted species, and at extreme densities, individuals of *P. aspera* probably benefited from this novel competitive environment in several ways (Fowler *et al.* 2006). At low density, shoot survival and growth probabilities could have increased as a direct response to reduced competition. Alternatively, at high densities, the increased ground cover of *P. aspera* could have mitigated the negative effect of moisture loss that is associated with fire (Henry *et al.* 2006).

Despite the likelihood that density and a fire disturbance can have interactive effects on populations (see e.g. Saether *et al.* 2000), investigations of these factors together are still uncommon. Because disturbance resulting from the effects of climate change is becoming more common, density and fire will probably interact at higher frequencies in the future. The results reported here suggest that factors driving changes in *λ* across populations can often be non-intuitive and context specific (see also Fowler *et al.* 2006) and that some well-established concepts of density effects will require further study.

## Sources of Funding

My work was funded, in part, by T. E. Miller, and by the Tall Timbers Research Station.

## Contributions by the Author

E.S.G. designed and executed all aspects of the experiment and wrote the manuscript.

## Conflicts of Interest Statement

None declared.

## Supporting Information

The following Supporting Information is available in the online version of this article –

**Figure S1.** Regressions of density and underlying abiotic factors across experimental plots at Tall Timbers Research Reserve, FL, USA.

**Table S1.** Population-projection matrices for *Pityopsis aspera* across densities. Life stages follow descriptions in Figure 1.

Additional Information

## References

[PLT041C1] Alvarez-Buylla ER, Slatkin M (1993). Finding confidence limits on population growth rates: Monte Carlo test of a simple analytic method. Oikos.

[PLT041C2] Amarasekare P (2004). Spatial variation and density-dependent dispersal in competitive coexistence. Proceedings of the Royal Society of London B Biological Sciences.

[PLT041C3] Antonovics J (1976). The nature of limits to natural selection. Annals of the Missouri Botanical Garden.

[PLT041C4] Antonovics J, Levin DA (1980). The ecological and genetic consequences of density-dependent regulation in plants. Annual Review of Ecology and Systematics.

[PLT041C5] Arnone JA, Jasoni RL, Lucchesi AJ, Larsen JD, Leger EA, Sherry RA, Luo YQ, Schimel DS, Verburg PSJ (2011). A climatically extreme year has large impacts on C-4 species in tallgrass prairie ecosystems but only minor effects on species richness and other plant functional groups. Journal of Ecology.

[PLT041C6] Ban S, Tenma H, Mori T, Nishimura K (2009). Effects of physical interference on life history shifts in *Daphnia pulex*. The Journal of Experimental Biology.

[PLT041C7] Bond WJ, van Wilgen BW (1996). Fire and plants.

[PLT041C8] Bowers FD (1972). A biosystematic study of Heterotheca section Pityopsis.

[PLT041C9] Brewer JS (2006). Long-term population changes of a fire-adapted plant subjected to different fire seasons. Natural Areas Journal.

[PLT041C10] Brewer JS (2008). Geographic variation in flowering responses to fire and season of clipping in a fire-adapted plant. The American Midland Naturalist.

[PLT041C11] Brewer JS, Platt WJ (1994a). The effects of fire season and herbivory on reproductive success of a clonal forb, *Pityopsis graminifolia*. Journal of Ecology.

[PLT041C12] Brewer JS, Platt WJ (1994b). Effects of fire season and soil fertility on clonal growth in a pyrophilic forb, *Pityopsis graminifolia* (Asteraceae). American Journal of Botany.

[PLT041C13] Buckley YM, Metcalf CJE, Cadotte MW, McMahon SM, Fukami T (2005). Density dependence in invasive plants: demography, herbivory, spread and evolution. Conceptual ecology and invasions biology.

[PLT041C14] Carpenter C (2005). The environmental control of plant species density on a Himalayan elevation gradient. Journal of Biogeography.

[PLT041C15] Casper BB (1996). Demographic consequences of drought in the herbaceous perennial *Cryptantha flava*: effects of density, associations with shrubs, and plant size. Oecologia.

[PLT041C16] Caswell H, Newman MC, Jagoe CH (1996). Demography meets ecotoxicology: untangling the population level effects of toxic substances. Ecotoxicology: a hierarchical treatment.

[PLT041C17] Caswell H (2001). Matrix population models: construction, analysis, and interpretation.

[PLT041C18] Caswell H (2010). Life table response experiment analysis of the stochastic growth rate. Journal of Ecology.

[PLT041C19] Chapin FS (1991). Integrated responses of plants to stress. BioScience.

[PLT041C20] Christensen NL, Mooney HA, Bonnicksen TM, Christensen NL, Lotan JE, Reiners WA (1981). Fire regimes in southeastern ecosystems. Fire regimes and ecosystem properties.

[PLT041C21] Dahlgren JP, Ehrlén J (2009). Linking environmental variation to population dynamics of a forest herb. Journal of Ecology.

[PLT041C22] Dalgleish HJ, Koons DN, Adler PB (2010). Can life-history traits predict the response of forb populations to changes in climate variability?. Journal of Ecology.

[PLT041C23] Daubenmire R (1968). Ecology of fire in grasslands. Advances in Ecological Research.

[PLT041C24] Davis HG, Taylor CM, Civille JC, Strong DR (2004). An Allee effect at the front of a plant invasion: Spartina in a Pacific estuary. Journal of Ecology.

[PLT041C25] de Kroon H, van Groenendael J, Ehrlen J (2000). Elasticities: a review of methods and model limitations. Ecology.

[PLT041C26] Dennis B, Desharnais RA, Cushing JM, Costantino RF (1995). Nonlinear demographic dynamics: mathematical models, statistical methods, and biological experiments. Ecological Monographs.

[PLT041C27] Doak DF, Gross K, Morris WF (2005). Understanding and predicting the effects of sparse data on demographic analyses. Ecology.

[PLT041C28] Evans MEK, Holsinger KE, Menges ES (2010). Fire, vital rates, and population viability: a hierarchical Bayesian analysis of the endangered Florida scrub mint. Ecological Monographs.

[PLT041C29] Feldman TS, Morris WF (2011). Higher survival at low density counteracts lower fecundity to obviate Allee effects in a perennial plant. Journal of Ecology.

[PLT041C30] Fetcher N, Shaver GR (1983). Life histories of tillers of *Eriophorum vaginatum* in relation to tundra disturbance. Journal of Ecology.

[PLT041C31] Fiske IJ, Bruna EM, Bolker BM (2008). Effects of sample size on estimates of population growth rates calculated with matrix models. PLoS ONE.

[PLT041C32] Fowler NL, Overath RD, Pease CM (2006). Detection of density dependence requires density manipulations and calculation of λ. Ecology.

[PLT041C33] Gadgil M, Solbrig OT (1972). Concept of r-selection and K-selection: evidence from wild flowers and some theoretical considerations. The American Naturalist.

[PLT041C34] Ghazoul J, Liston KA, Boyle TJB (2012). Disturbance-induced density-dependent seed set in *Shorea siamensis* (Dipterocarpaceae), a tropical forest tree. Journal of Ecology.

[PLT041C35] Goldberg DE (1987). Neighborhood competition in an old-field plant community. Ecology.

[PLT041C36] Goldberg DE, Grace J, Tilman D (1990). Components of resource competition in plant communities. Perspectives on plant competition.

[PLT041C37] Goldberg DE, Turkington R, Olsvig-Whittaker L, Dyer AR (2001). Density dependence in an annual plant community: variation among life history stages. Ecological Monographs.

[PLT041C38] Gowe AK, Brewer JS (2005). The evolution of fire-dependent flowering in goldenasters (*Pityopsis* spp.). Journal of the Torrey Botanical Society.

[PLT041C39] Grant A (1998). Population consequences of chronic toxicity: incorporation density dependence into the analysis of life table response experiments. Ecological Modelling.

[PLT041C40] Greene CM, Stamps JA (2001). Habitat selection at low population densities. Ecology.

[PLT041C41] Grime JP (1979). Plant strategies, vegetation processes, and ecosystem properties.

[PLT041C42] Gross K, Lockwood JR, Frost CC, Morris WF (1998). Modeling controlled burning and trampling reduction for conservation of *Hudsonia montana*. Conservation Biology.

[PLT041C43] Hackney EE, McGraw JB (2001). Experimental demonstration of an Allee effect in American ginseng. Conservation Biology.

[PLT041C44] Hartnett DC, Bazzaz FA (1985). The genet and ramet population-dynamics of *Solidago canadensis* in an abandoned field. Journal of Ecology.

[PLT041C45] Hastie TJ, Tibshirani RJ (1990). Generalized additive models.

[PLT041C46] Henry HAL, Chiariello NR, Vitousek PM, Mooney HA, Field CB (2006). Interactive effects of fire, elevated carbon dioxide, nitrogen deposition, and precipitation on a California annual grassland. Ecosystems.

[PLT041C47] Hixon MA, Pacala SW, Sandin SA (2002). Population regulation: historical context and contemporary challenges of open vs. closed systems. Ecology.

[PLT041C48] Hobbs RJ, Mooney HA (1985). Vegetative regrowth following cutting in the shrub *Baccharis pilularis* spp. *consanguinea* (DC) C.B. Wolf. American Journal of Botany.

[PLT041C49] Holt RD (1987). Population dynamics and evolutionary processes: the manifold roles of habitat selection. Evolutionary Ecology.

[PLT041C50] Holt RD, Keitt TH, Lewis MA, Maurer BA, Taper ML (2005). Theoretical models of species' borders: single species approaches. Oikos.

[PLT041C51] Hulbert LC (1988). Causes of fire effects in tallgrass prairie. Ecology.

[PLT041C52] Hutchings MJ (1983). Ecology's law in search of a theory. New Scientist.

[PLT041C53] Jesson L, Kelly D, Sparrow A (2000). The importance of dispersal, disturbance, and competition for exotic plant invasions in Arthur's Pass National Park, New Zealand. New Zealand Journal of Botany.

[PLT041C54] Johnson MTJ (2008). Bottom-up effects of plant genotype on aphids, ants, and predators. Ecology.

[PLT041C55] Kaye TN, Pendergrass KL, Finley K, Kauffman JB (2001). The effect of fire on the population viability of an endangered prairie plant. Ecological Applications.

[PLT041C56] Kesler HC, Trusty JL, Hermann SM, Guyer C (2008). Demographic responses of *Pinguicula ionantha* to prescribed fire: a regression-design LTRE approach. Oecologia.

[PLT041C57] Lesica P (1999). Effects of fire on the demography of the endangered, geophytic herb *Silene spaldingii* (Caryophyllaceae). American Journal of Botany.

[PLT041C58] Levine JM, Adler PA, Yelenik SG (2004). A meta-analysis of biotic resistance to exotic plant invasions. Ecology Letters.

[PLT041C59] Linke-Gamenick I, Forbes VE, Sibly RM (1999). Density-dependent effects of a toxicant on life-history traits and population dynamics of a capitellid polychaete. Marine Ecology Progress Series.

[PLT041C60] Liu H, Menges ES, Snyder JR, Koptur S, Ross MS (2005). Effects of fire intensity on vital rates of an endemic herb of the Florida Keys, USA. Natural Areas Journal.

[PLT041C61] Lloyd AH, Wilson AE, Fastie CL, Landis RM (2005). Population dynamics of black spruce and white spruce near the arctic tree line in the southern Brooks Range, Alaska. Canadian Journal of Forest Research.

[PLT041C62] Loehle C (1987). Partitioning of reproductive effort in clonal plants: a benefit-cost model. Oikos.

[PLT041C63] Logofet DO (1993). Matrices and graphs: stability problems in mathematical ecology.

[PLT041C64] Menges ES, Dolan RW (1998). Demographic viability of populations of *Silene regia* in Midwestern prairies: relationships with fire management, genetic variation, geographic location, population size and isolation. Journal of Ecology.

[PLT041C65] Menges ES, Weedley CW, Clarke GL, Smith SA (2011). Effects of hurricanes on rare plant demography in fire-controlled ecosystems. Biotropica.

[PLT041C66] Miller TE (1996). On quantifying the intensity of competition across gradients. Ecology.

[PLT041C67] Myers RL, Ewel JJ (1990). The ecosystems of Florida.

[PLT041C68] Ogden J (1974). The reproductive strategy of higher plants: II. The reproductive strategy of *Tussilago farfara* L. Journal of Ecology.

[PLT041C69] Oli MK, Slade NA, Dobson FS (2001). Effect of density reduction on Uinta ground squirrels: analysis of life table response experiments. Ecology.

[PLT041C70] Rayburn AP, Schupp EW (2013). Effects of community- and neighbourhood-scale spatial patterns on semi-arid perennial grassland community dynamics. Oecologia.

[PLT041C71] Reid AM, Robertson KM, Hmielowski TL (2012). Predicting litter and live herb fuel consumption during prescribed fires in native and old-field upland pine communities of the southeastern United States. Canadian Journal of Forest Research.

[PLT041C72] Rey PJ, Alcantara JM, Valera F, Sanchez-Lafuente AM, Garrido JL, Ramirez JM, Manzaneda AJ (2004). Seedling establishment in *Olea europaea:* seed size and microhabitat affect growth and survival. Ecoscience.

[PLT041C73] Saether BE, Engen S, Lande R, Arcese P, Smith JNM (2000). Estimating the time to extinction in an island population of song sparrows. Proceedings of the Royal Society of London B Biological Sciences.

[PLT041C74] Schemske DW, Husband BC, Ruckelshaus MH, Goodwillie C, Parker CM, Bishop JG (1994). Evaluating approaches to the conservation of rare and endangered plants. Ecology.

[PLT041C75] Sherry RA, Arnone JA, Johnson DW, Schimel DS, Verburg PS, Luo YQ (2012). Carry over from previous year environmental conditions alters dominance hierarchy in a prairie plant community. Journal of Plant Ecology.

[PLT041C76] Sibly RM, Barker D, Denham MC, Hone J, Pagel M (2005). On the regulation of populations of mammals, birds, fish, and insects. Science.

[PLT041C77] Stokes KE, Bullock JM, Watkinson AR (2004). Population dynamics across a parapatric range boundary: *Ulex gallii* and *Ulex minor*. Journal of Ecology.

[PLT041C78] Thomas AG, Dale HM (1975). The role of seed reproduction in the dynamics of established populations of *Hieracium floribundum* and a comparison with that of vegetative reproduction. Canadian Journal of Botany.

[PLT041C79] Truscott AM, Palmer SCF, Soulsby C, Hulme PE (2008). Assessing the vulnerability of riparian vegetation to invasion by *Mimulus guttatus*: relative importance of biotic and abiotic variables in determining species occurrence and abundance. Diversity and Distributions.

[PLT041C80] Wang X, He HS, Li X (2007). The long-term effects of fire suppression and reforestation on a forest landscape in Northeastern China after a catastrophic wildfire. Landscape and Urban Planning.

[PLT041C81] Weekley CW, Mendes ES (2012). Burning creates contrasting demographic patterns in *Polygala lewtonii* (Polygalaceae): a cradle-to-grave analysis of multiple cohorts in a perennial herb. Australian Journal of Botany.

