# Supplementary material for: Effects of density and fire on the vital rates and population growth of a perennial goldenaster
Source: AoB Plants. 2013 Sep 9;5:plt041. doi: 10.1093/aobpla/plt041 (PMC4455675; doi:10.1093/aobpla/plt041)
Supplement: Additional Information [file supp_5_plt041_index.html]

Additional Information 

# Effects of density and fire on the vital rates and population growth of a perennial goldenaster

## Additional Information

Additional Information

**Files in this Data Supplement:**

- Additional Information - doc file
