# Supplementary material for: Effects of density and fire on the vital rates and population growth of a perennial goldenaster
Source: AoB Plants. 2013 Sep 9;5:plt041. doi: 10.1093/aobpla/plt041 (PMC4455675; doi:10.1093/aobpla/plt041)
Supplement: Additional Information [file supp_plt041_plt041supp.doc]

**Figure S1. Regressions of density and underlying abiotic factors.**

| 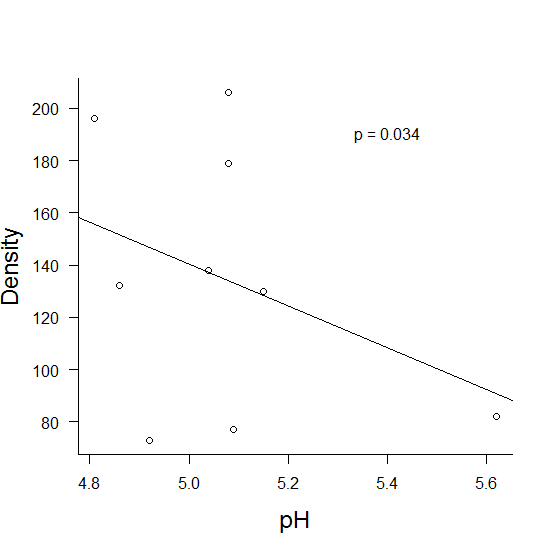 | 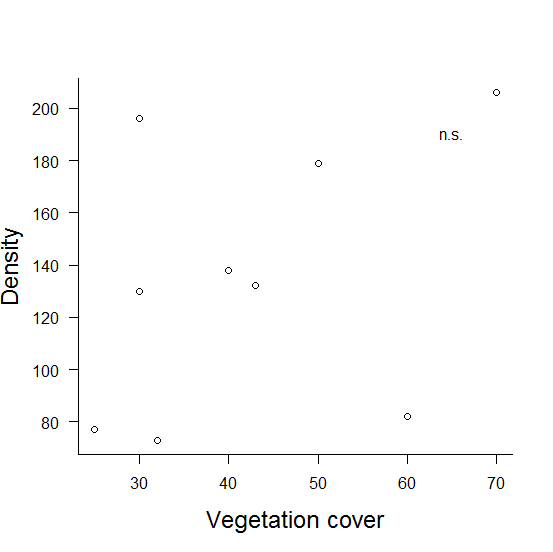 |
| --- | --- |
| 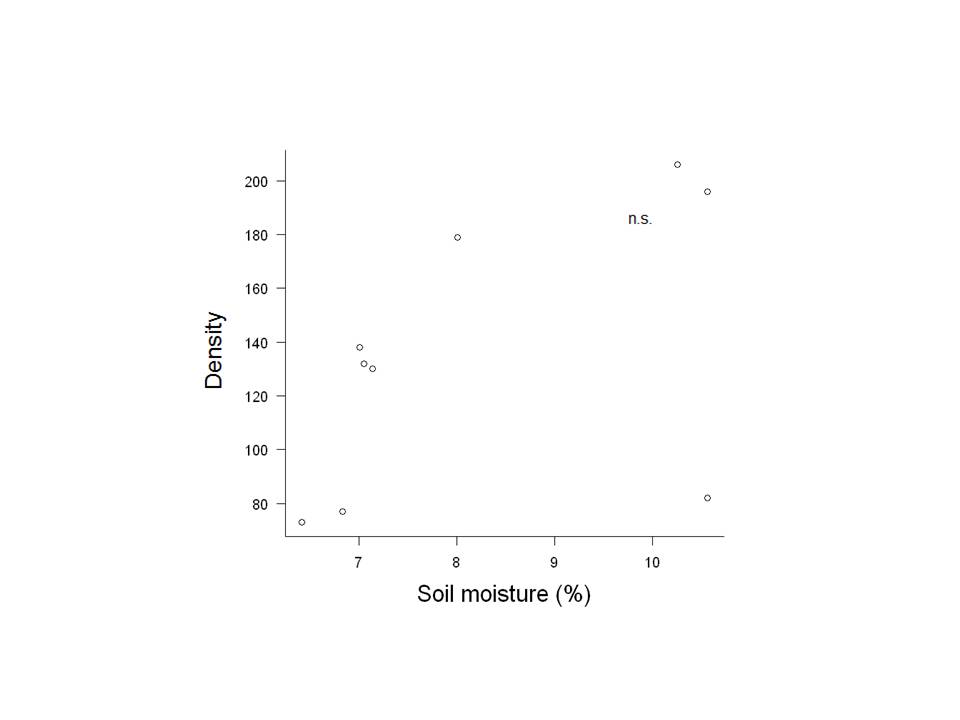 | 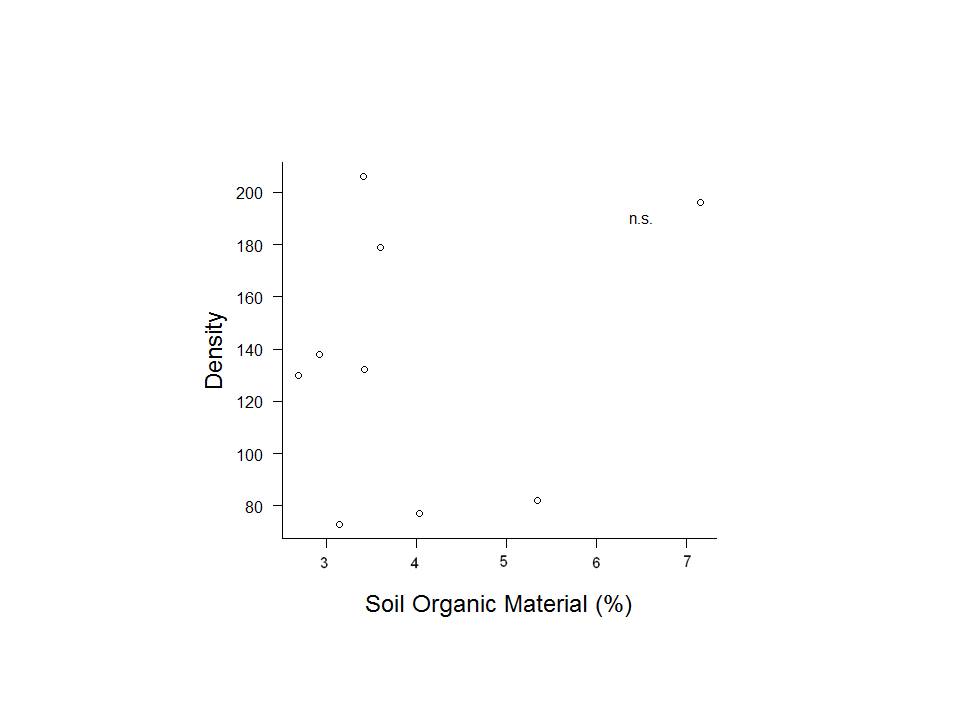 |
| 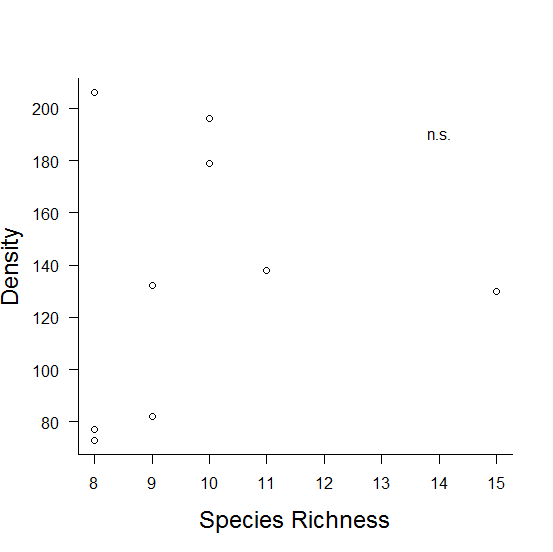 |  |

**Table S1.** Population projection matrices for *Pityopsis aspera* across densities. Life stages follow descriptions in figure 1: s = Rosette first-year; j = Flowering first-year; r = Rosette adult; f = Flowering adult. Values in parentheses indicate asexual reproduction.

|  | **2009 – 2010** | **2010 - 2011** |
| --- | --- | --- |
|  |  |  |
| **Density = 71** | |  | *s* | *j* | *r* | *f* | | --- | --- | --- | --- | --- | | *s* | 0 | 0.347 | 0 | 1.480 | | *j* | 0 | 0.047 | 0 | 0.199 | | *r* | 0.166 | 0.25 | 0.435 (0.261) | 0.512 (0.293) | | *f* | 0 | 0 | 0.087 | 0.049 (0.024) | | |  | *s* | *j* | *r* | *f* | | --- | --- | --- | --- | --- | | *s* | 0 | 0.873 | 0 | 6.571 | | *j* | 0 | 0.266 | 0 | 2.000 | | *r* | 0.205 | 0.077 | 0.471 (0.098) | 0.333 | | *f* | 0.026 | 0.115 | 0.059 | 0.000 | |
|  |  |  |
| **Density = 77** | |  | *s* | *j* | *r* | *f* | | --- | --- | --- | --- | --- | | *s* | 0 | 0.191 | 0 | 1.460 | | *j* | 0 | 0.015 | 0 | 0.114 | | *r* | 0.194 | 0.429 | 0.368 (0.285) | 0.357 (0.262) | | *f* | 0 | 0 | 0.053 (0.026) | 0.024 | | |  | *s* | *j* | *r* | *f* | | --- | --- | --- | --- | --- | | *s* | 0 | 0 | 0 | 10.809 | | *j* | 0 | 0 | 0 | 1.544 | | *r* | 0.052 | 0 | 0.319 (0.125) | 0 | | *f* | 0.04 | 0 | 0.085 (0.208) | 0.750 (0.250) | |
|  |  |  |
| **Density = 91** | |  | *s* | *j* | *r* | *f* | | --- | --- | --- | --- | --- | | *s* | 0 | 0.063 | 0 | 0.128 | | *j* | 0 | 0 | 0 | 0 | | *r* | 0.250 | 0.5 | 0.455 (0.273) | 0.447 (0.145) | | *f* | 0 | 0 | 0.091 (0.045) | 0.079 (0.079) | | |  | *s* | *j* | *r* | *f* | | --- | --- | --- | --- | --- | | *s* | 0 | 0.463 | 0 | 5.721 | | *j* | 0 | 0.046 | 0 | 0.550 | | *r* | 0.050 | 0 | 0.180 (0.02) | 0 | | *f* | 0 | 0.111 | 0.140 | 0.250 | |
|  |  |  |
| **Density = 130** | |  | *s* | *j* | *r* | *f* | | --- | --- | --- | --- | --- | | *s* | 0 | 0.692 | 0 | 1.307 | | *j* | 0 | 0.183 | 0 | 0.345 | | *r* | 0.280 | 0.571 | 0.449 (0.236) | 0.471 (0.129) | | *f* | 0.065 | 0.048 | 0.090 (0.022) | 0.229 (0.043) | | |  | *s* | *j* | *r* | *f* | | --- | --- | --- | --- | --- | | *s* | 0 | 0.419 | 0 | 3.208 | | *j* | 0 | 0.205 | 0 | 1.568 | | *r* | 0.044 | 0.050 | 0.352 (0.007) | 0.250 (0.028) | | *f* | 0.011 | 0.100 | 0.113 (0.014) | 0.028 | |
|  |  |  |
| **Density = 132** | |  | *s* | *j* | *r* | *f* | | --- | --- | --- | --- | --- | | *s* | 0 | 0.033 | 0 | 0.170 | | *j* | 0 | 0.007 | 0 | 0.036 | | *r* | 0.122 | 0.219 | 0.250 (0.114) | 0.509 (0.091) | | *f* | 0.051 | 0.125 | 0.417 (0.083) | 0.118 (0.064) | | |  | *s* | *j* | *r* | *f* | | --- | --- | --- | --- | --- | | *s* | 0 | 0.322 | 0 | 7.303 | | *j* | 0 | 0.01 | 0 | 0.227 | | *r* | 0.105 | 0.174 | 0.186 (0.058) | 0.028 (0.083) | | *f* | 0.032 | 0.130 | 0.279 (0.023) | 0.222 (0.056) | |
|  |  |  |
| **Density = 138** | |  | *s* | *j* | *r* | *f* | | --- | --- | --- | --- | --- | | *s* | 0 | 0.166 | 0 | 0.622 | | *j* | 0 | 0.050 | 0 | 0.189 | | *r* | 0.302 | 0.474 | 0.373 (0.237) | 0.529 (0.176) | | *f* | 0.038 | 0 | 0.034 (0.017) | 0.047 (0.059) | | |  | *s* | *j* | *r* | *f* | | --- | --- | --- | --- | --- | | *s* | 0 | 0 | 0 | 3.625 | | *j* | 0 | 0 | 0 | 2.875 | | *r* | 0.036 | 0.056 | 0.280 (0.022) | 0.375 | | *f* | 0 | 0 | 0.118 | 0 | |
|  |  |  |
| **Density = 179** | |  | *s* | *j* | *r* | *f* | | --- | --- | --- | --- | --- | | *s* | 0 | 0.493 | 0 | 1.438 | | *j* | 0 | 0.070 | 0 | 0.203 | | *r* | 0.426 | 0.375 | 0.456 (0.158) | 0.627 (0.082) | | *f* | 0.059 | 0.083 | 0.140 (0.018) | 0.045 (0.118) | | |  | *s* | *j* | *r* | *f* | | --- | --- | --- | --- | --- | | *s* | 0 | 0.888 | 0 | 13.195 | | *j* | 0 | 0.066 | 0 | 0.977 | | *r* | 0.024 | 0.030 | 0.406 (0.051) | 0.238 (0.235) | | *f* | 0.024 | 0.091 | 0.181 (0.316) | 0.143 (0.118) | |
|  |  |  |
| **Density = 196** | |  | *s* | *j* | *r* | *f* | | --- | --- | --- | --- | --- | | *s* | 0 | 0.154 | 0 | 0.933 | | *j* | 0 | 0.049 | 0 | 0.295 | | *r* | 0.462 | 0.391 | 0.638 (0.138) | 0.582 (0.173) | | *f* | 0.038 | 0.043 | 0.074 (0.021) | 0.051 (0.071) | | |  | *s* | *j* | *r* | *f* | | --- | --- | --- | --- | --- | | *s* | 0 | 3.281 | 0 | 4.386 | | *j* | 0 | 0.174 | 0 | 0.233 | | *r* | 0.568 | 0.412 | 0.373 (0.089) | 0.222 (0.565) | | *f* | 0.045 | 0.294 | 0.012 (0.683) | 0.167 (0.182) | |
|  | | |
| **Density = 206** | |  | *s* | *j* | *r* | *f* | | --- | --- | --- | --- | --- | | *s* | 0 | 0.173 | 0 | 0.477 | | *j* | 0 | 0.028 | 0 | 0.078 | | *r* | 0.617 | 0.636 | 0.400 (0.150) | 0.516 (0.145) | | *f* | 0.050 | 0.091 | 0.250 (0.050) | 0.121 (0.056) | | |  | *s* | *j* | *r* | *f* | | --- | --- | --- | --- | --- | | *s* | 0 | 2.214 | 0 | 9.778 | | *j* | 0 | 0.162 | 0 | 0.717 | | *r* | 0.333 | 0.333 | 0.269 (0.194) | 0.222 (0.083) | | *f* | 0 | 0.667 | 0.283 (0.343) | 0.444 (0.306) | |
